# Comprehensive stabilization mechanism of electron-beam irradiated polyacrylonitrile fibers to shorten the conventional thermal treatment

**DOI:** 10.1038/srep27330

**Published:** 2016-06-28

**Authors:** Sejoon Park, Seung Hwa Yoo, Ha Ri Kang, Seong Mu Jo, Han-Ik Joh, Sungho Lee

**Affiliations:** 1 Carbon Convergence Materials Research Center, Institute of Advanced Composite Materials, Korea Institute of Science and Technology, 92, Chudong-ro, Bongdong-eup, Wanju-gun, Jeollabuk-do 565-905, Republic of Korea

## Abstract

An electron beam was irradiated on polyacrylonitrile (PAN) fibers prior to thermal stabilization. The electron-beam irradiation effectively shortened the thermal stabilization process by one fourth compared with the conventional thermal stabilization process. A comprehensive mechanistic study was conducted regarding this shortening of the thermal stabilization by electron-beam irradiation. Various species of chain radicals were produced in PAN fibers by electron-beam irradiation and existed for a relatively long duration, as observed by electron spin resonance spectroscopy. Subsequently, these radicals were gradually oxidized to peroxy radicals in the presence of oxygen under storage or heating. We found that these peroxy radicals (CO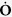) enabled such an effective shortcut of thermal stabilization by acting as intermolecular cross-linking and partial aromatization points in the low temperature range (100–130 °C) and as earlier initiation seeds of successive cyclization reactions in the next temperature range (>130–140 °C) of thermal stabilization. Finally, even at a low irradiation dose (200 kGy), followed by a short heat treatment (230 °C for 30 min), the PAN fibers were sufficiently stabilized to produce carbon fibers with tensile strength and modulus of 2.3 and 216 GPa, respectively, after carbonization.

Carbon fiber (CF) has attracted great interest in both research and industrial fields because of its excellent mechanical properties, especially its tensile strength^1^,^2^. Currently, carbon fibers provide reinforcement in composites with suitable resins^3^, which are applied as structural materials in aircraft, spacecraft, sports equipment, etc^4^,^5^,^6^. However, the high cost of CFs hinders their application in more diverse fields. Therefore, much effort has been made to reduce the production costs of CFs, while maintaining its tensile strength, to broaden its applicable fields to automobiles, construction, oil drilling, infrastructure industry, etc^7^,^8^,^9^. To produce CFs, the conventional fabrication process includes three main steps: fabrication of precursor polymer fibers by spinning, thermal stabilization, and thermal carbonization^10^,^11^. The thermal stabilization process is a long and tedious heating process, which is the most energy- and time-consuming step. For these reasons, efforts have been made to reduce the energy and time consumption of the thermal stabilization process^12^,^13^,^14^.

Recently, pioneering works have been performed by researchers to shorten the thermal stabilization time by ionizing radiation (gamma-ray or electron-beam) or plasma treatment prior to or during stabilization^14^,^15^,^16^,^17^. Among these technologies, electron-beam irradiation was found to be an especially powerful tool for treating polymer fibers. Specifically, electron irradiation can be conducted at higher dose rates than gamma-ray irradiation, consequently shortening the treatment time^18^,^19^. Additionally, the penetration depth of the energetic electrons is sufficient to treat bundles of fibers. However, despite these advantages, only a few studies conducted by diverse electron irradiation and precursor fiber conditions have been reported^16^,^17^,^20^,^21^,^22^,^23^,^24^. In general, electron irradiation can induce two main effects when applied to polymers: radiolysis of molecular bonds and rise of local temperature by energetic electron bombardment. In the case of radiolysis, energetic electrons can transfer some of their energy to the covalent bond electrons in the polymers, which is sufficient to break these covalent bonds and produce various radicals. Alternatively, the transferred energy can also excite the covalent bond electrons, releasing energy mainly as heat by consequent de-excitation. Because polymers are thermal insulators, this heat may not be efficiently dissipated, possibly resulting in local temperature rise within the material^25^,^26^. Therefore, at low current density, one can exclude the local heating effect and solely examine the radiolysis effect. After the pioneering study by J. Dietrich in 1996^17^, which showed that electron-beam irradiation can significantly reduce the thermal stabilization time of polyacrylonitrile (PAN) fibers, no studies have been reported on the electron irradiation of PAN fibers with low beam current and low dose in air conditions. Several reports have described electron irradiation of PAN fibers in which the irradiation induced cross-linking and cyclization reactions^16^,^20^,^21^,^22^,^23^,^24^. These reports are commonly conducted at high beam current, which may cause local temperature rise within the fiber even though the irradiation was conducted at room temperature. Furthermore, although studies have been conducted to observe the radicals formed by gamma-ray irradiation in PAN fibers^15^, there have been no reports in the literature that observe the radicals formed by electron-beam irradiation since 1996. Therefore, the role of electron irradiation prior to thermal stabilization as well as the mechanism of shortening the thermal stabilization time by electron irradiation on PAN fibers have yet to be revealed.

In this study, electron-beam irradiation was performed on PAN fibers to reduce the thermal stabilization time. The electron irradiation was conducted at low beam current (1 mA) with very low beam current density (1.36 μA cm^−2^) and dose (200–1500 kGy). The effects of electron irradiation on thermal stabilization of PAN fibers were carefully and thoroughly analyzed, and a reasonable mechanism was deduced based on the analysis results.

## Results and Discussion

### Electron-beam irradiation of PAN fibers

**Figure 1:**
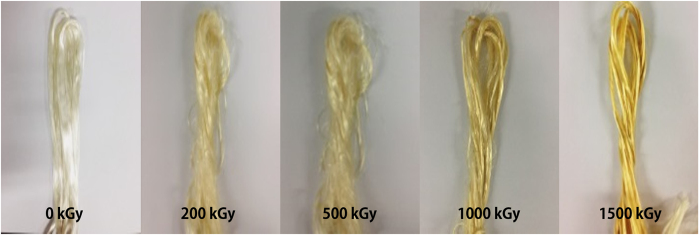
Photographs of PAN fibers irradiated at various electron doses.

The photographs of PAN fibers irradiated at various electron doses (0, 200, 500, 1000, and 1500 kGy) are shown in Fig. 1. The apparent color of the fibers gradually changed from shiny white of the non-irradiated fibers to pale yellow of the irradiated fibers as the electron dose was increased, and finally to bright yellow at 1500 kGy electron dose. The color change of polymeric materials by irradiation with ionizing radiation is caused by the formation of conjugated double bonds (C=C)_n_ or color centers associated with radical species trapped within glassy polymer matrices, as previously reported in the literature^27^,^28^.

**Figure 2:**
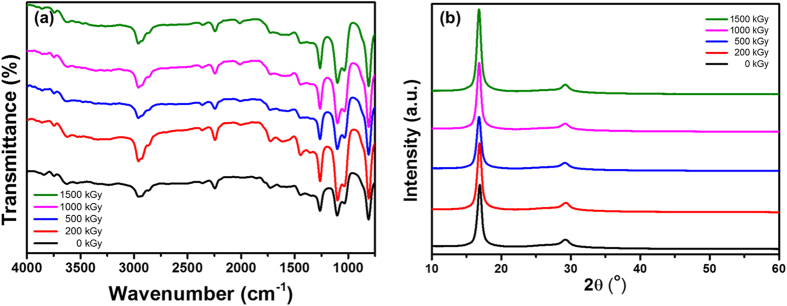
(**a**) FT-IR spectra and (**b**) XRD patterns of PAN fibers irradiated at various electron doses.

Figure 2a shows the FT-IR spectra of the electron-irradiated PAN fibers. The non-irradiated PAN fibers showed several molecular vibrations originating from their constituents. The stretching of the alkane C-H and the nitrile C≡N were observed at 2935 and 2245 cm^−1^, respectively. These vibrations are typical features of the PAN homopolymer, which consists only of acrylonitrile (AN) monomers^29^,^30^. Along with the vibrations from the AN monomer, stretching of the carbonyl C=O, aldehyde C-H, and carboxylic acid O-H were also observed at 1728, 2862, and 2300–3500 cm^−1^, respectively. Therefore, it is evident that the PAN fiber used in this study is a copolymer of AN and comonomer containing a carboxylic acid group^31^. Interestingly, although it is well known that energetic electron irradiation can easily cause bond cleavage of polymeric materials, no significant changes were observed by FT-IR spectroscopy until an electron dose of 1500 kGy.

In modern technology, PAN fibers are commonly produced by wet or dry spinning, followed by thorough washing of residual solvents and simultaneous gradual drawing and heat treatment^32^. During the drawing process, the orientation of the polymer chains is aligned with the drawing direction, forming a highly crystalline structure. These crystalline structures of the PAN fibers were verified by XRD patterns, as shown in Fig. 2b. The diffraction peaks centered at 2θ = 16.8 and 29.2° correspond to the (100) and (110) crystallographic planes of the PAN fiber, respectively. Not surprisingly, no significant changes were observed in the XRD patterns of electron-irradiated PAN fibers, which is consistent with the FT-IR analysis.

**Figure 3:**
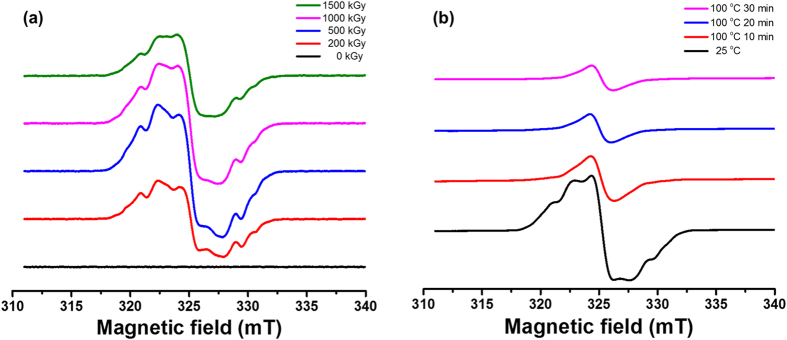
ESR spectra of PAN fibers irradiated at (**a**) various electron doses and (**b**) 200 kGy with post-heating at 100 °C for 10, 20, and 30 min.

When polymers are subjected to irradiation with ionizing radiation, various radicals can be formed as a result of the cleavage of molecular bonds within the material. These radicals, which are formed by radiolysis, were monitored by ESR spectroscopy^33^,^34^,^35^. The ESR spectra of the electron-irradiated PAN fibers are shown in Fig. 3a. Regardless of electron dose, all of the irradiated PAN fibers showed a typical spectrum that exhibited a hyperfine structure. These structures originate from the superposition of the spectra of several radicals produced by the electron irradiation, which consist of a hyperfine structure of alkyl radicals formed by C-H bond cleavage of (1) secondary (-CH(CN)-ĊH-CH(CN)-) and (2) tertiary (-CH_2_-Ċ(CN)-CH_2_-) carbons, (3) allyl radicals formed by the cleavage of two neighboring C-H bonds and (4) a broad singlet structure of polyenyl radicals formed by cleavage of multiple C-H bonds of neighboring hydrogens in the polymer backbone^35^. In the case of the alkyl radical (1), two β protons (H_β_) induce the triplet lines with hyperfine splitting, whereas one α proton (H_α_) induces the doublet lines to yield a six-line spectrum. For the alkyl radical (2), four H_β_ induce the quintet lines with hyperfine splitting to yield a five-line spectrum^34^,^35^,^36^. For the allyl radicals, the unpaired electron is delocalized and stabilized by resonating in the π-orbitals of three adjacent carbon atoms, four H_β_ induce the quintet lines with hyperfine splitting, and one H_α_ induces the doublet lines to yield a ten-line spectrum^35^. For the polyenyl radicals, the unpaired electron is delocalized by bond resonance on several adjacent carbon atoms, which makes the hyperfine splitting narrow enough to show a broad singlet spectrum^37^,^38^. The hyperfine splitting constant (*a*) for H_α_ and H_β_ is estimated to be 1.9–2.2 mT and 2.8–3.6 mT, respectively, with line-widths of 0.7–1.5 mT in our study, which reasonably agrees with the values of alkyl and allyl radicals of gamma and electron irradiated polyethylene and PAN in other reports^34^,^35^,^36^ (Figures S1 and S2).

**Figure 4:**
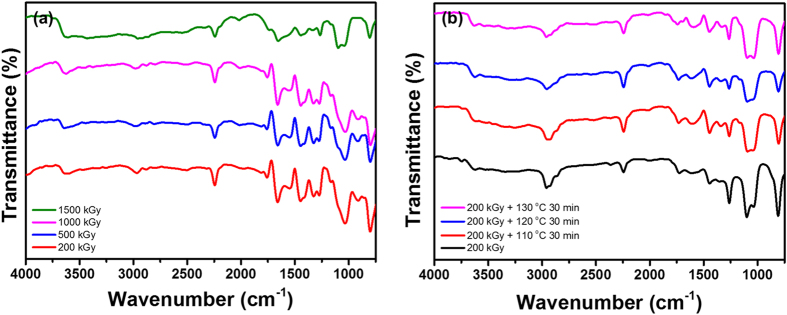
FT-IR spectra of PAN fibers irradiated at various electron doses after (**a**) dissolving in DMF for 12 h followed by vacuum drying overnight and (**b**) heating at 110, 120, and 130 °C for 30 min in air.

The broad singlet is assumed to be the polyenyl radical rather than the polyimine radical, which was previously reported by W. Liu^34^. Based on our FT-IR analysis, no formation of a C=N bond was observed after dissolving the electron-irradiated PAN fibers in DMF (Fig. 4a), indicating that polyimine radicals may not be the main product under our experimental conditions. Note that the g-values of the radicals were estimated to be 2.00243–2.00258 for a 200–1500 kGy electron dose. These values are slightly lower than the values of alkyl and allyl radicals (2.0026–2.0027) previously reported in the literature^39^,^40^. We presume that this result is attributed to the shift of the neighboring conjugated π-electrons proposed by S. Hasegawa^38^. After prolonged storage or heating at 100 °C under air, the ESR spectra gradually transformed to a broad singlet accompanied by a decrease in intensity. However, the g-values increased from 2.00251 to 2.00392 (Figures S3 and S4). These observations are suggested to originate from the intrinsic decay of alkyl, allyl, and polyenyl radicals^41^ and the gradual transformation to peroxy radicals, as shown in Fig. 3b^35^,^36^,^42^. In addition, the oxygen content of irradiated PAN fibers increased as the electron dose and storage time in air increased, which also supports the fact that oxygen is gradually increasing in irradiated PAN fibers (Figure S5). Therefore, based on the ESR analysis, we suggest that electron irradiation produced various radicals in the PAN fibers, which gradually change to peroxy radicals in the presence of oxygen.

It is interesting to note that even though radicals are formed in the PAN fiber by electron irradiation, no chemical reactions occurred to form new molecular bonds or induce changes in the crystalline structure. Several literature studies have reported the cross-linking and cyclization reaction of PAN molecules by irradiating with ionizing radiation, which was analyzed by FT-IR spectroscopy^15^,^16^,^24^,^43^,^44^. However, in this study, no evidence of cross-linking or cyclization reactions was observed by FT-IR analysis (Fig. 2a). We assume that the radicals formed along the polymer chains are trapped and fixed in the crystalline lattice as a result of the high crystallinity of PAN fibers. Furthermore, electron irradiation was conducted at room temperature, which is much lower than the glass transition temperature (T_g_) of PAN (~100 °C). Under these conditions, the mobility of polymer chains is restricted so that neither radical recombination can occur to form new chemical bonds nor electron irradiation can induce changes in the crystalline structure, which is supported by previous FT-IR and XRD analyses as mentioned above.

### Cross-linking and partial aromatization of electron-irradiated PAN fibers

To verify this presumption, the mobility of radicals on the polymer chains was provided by dissolving the electron-irradiated PAN fibers in DMF or heating above T_g_ under an air atmosphere. Figure 4a shows the FT-IR spectra of the electron-irradiated PAN fiber residuals after dissolving in DMF, followed by thorough drying. For non-irradiated PAN fibers, no residuals remained after dissolving in DMF. It is noteworthy that considerable changes in the FT-IR spectra are shown for the residuals of electron-irradiated PAN fibers. The appearance of new vibration modes at 1662 and 1450 cm^−1^ (stretching of alkene C=C and aromatic C-C, respectively) and significant development of molecular vibrations at 1327, 1169, and 1038 cm^−1^ (two stretching modes of ester acyl C-O and alkoxy C-O, respectively) are observed. However, along with the development of these vibrations, vibrations at 1265, 1716, and a broad range at 2300–3500 cm^−1^ (stretching of carbonyl acid C-O, C=O, and O-H, respectively) decreased for the electron-irradiated PAN fibers. Therefore, we suggest that alkyl, allyl, and polyenyl radicals produced by dehydrogenation and scission of irradiated-PAN polymer chains recombine with each other to form intermolecular cross-linking by C-C bonds as well as cross-linking through C-O-C bonds. These are expected to originate from the recombination of radicals produced by dehydrogenation of carboxylic acid comonomers and peroxy radicals (CO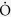), which were gradually formed from radicals initially produced by irradiation and further exposed to air.

As mentioned above, after dissolving the electron-irradiated fibers in DMF, followed by subsequent drying, a certain amount of residual was formed as a gel. The gelation of the polymers is a result of intermolecular cross-linking by recombination of radicals, which were formed by radiolysis^26^. The gel contents increased to 72, 90, 96, and 100% as the electron dose increased to 200, 500, 1000, and 1500 kGy, respectively. It is evident that intermolecular cross-linking occurred when sufficient mobility was provided to the electron-irradiated polymer chains. It is noteworthy that peroxy radicals can induce cross-linking between polymer chains. Furthermore, these radicals play a significant role in other reactions to reduce the stabilization time of PAN fibers. On the other hand, irradiated PAN fibers were heated to 110, 120, and 130 °C for 30 min in air to observe the reaction of the radicals.

As shown in Fig. 4b, the vibrations at 1165 cm^−1^ (esters acyl C-O) and 1697 cm^−1^ (acid C=O in ring) increased as the electron dose increased. In addition, the vibrations at 1614 cm^−1^ (conjugated C-C) gradually shifted to 1590 cm^−1^ (aromatic C-C) (detailed in Figure S6)^45^. Based on these facts, intermolecular cross-links were found to form from C-C and C-O-C bonds and aromatic structures were partially formed as a result of further dehydrogenation.

**Figure 5:**
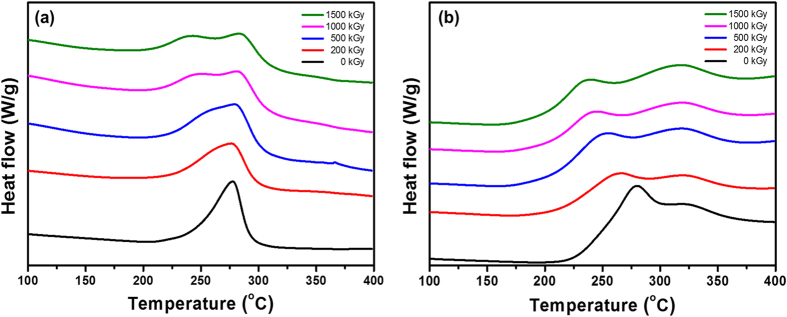
DSC curves of PAN fibers irradiated at various electron doses measured in (**a**) N_2_ and (**b**) air atmosphere.

**Figure 6:**
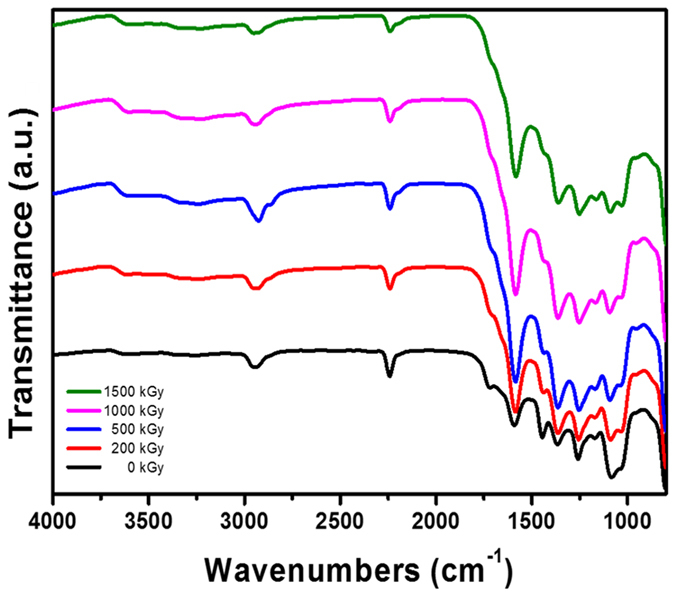
FT-IR spectra of PAN fibers heated at 230 °C for 30 min after irradiation at various electron doses.

To take a closer look at the thermal behavior of irradiated PAN fibers, DSC was conducted in N_2_ and air atmospheres (Fig. 5). First, in N_2_ atmosphere, the non-irradiated PAN fiber showed a sharp exothermal peak at 277.2 °C with a small tail below this temperature. The strong exothermic reaction centered at 277.2 °C is assigned as the cyclization reaction of the PAN homopolymer through a radical mechanism^46^. Therefore, the weak exothermic reaction below this temperature, which corresponds to the small tail, is assumed to be the cyclization of PAN initiated by the comonomers present in the PAN fiber used in this study. It is well known that a comonomer, such as itaconic acid (IA), can initiate the cyclization of PAN by an ionic mechanism^31^,^47^,^48^,^49^. In addition, precedent works have shown that comonomers, such as methyl acrylate and methyl methacrylate, can initiate the cyclization of PAN by nucleophilic or electrophilic attack of the carbon of the nitrile group^17^,^49^,^50^. As verified by the FT-IR analysis, one of the comonomers contains a carboxylic acid group, indicating that IA is present in the PAN fibers used in this study. Additionally, the cyclization reactions induced by radical and ionic mechanisms are observed by heating the non-irradiated PAN fiber at 230 °C for 30 min in Fig. 6.

In agreement with prior reports^23^,^24^, the onset temperature (T_onset_) of the exothermic reaction decreased as the electron dose was increased. Furthermore, the tail peak shifted to lower temperature as the electron dose was increased. The sharp peak, which is positioned at 277.2 °C for non-irradiated PAN fiber, shifted to slightly higher temperature as the electron dose was increased. Accompanied by temperature shifts, the peaks broadened as the electron dose was increased. These observations have been reported by several researchers but with ambiguous explanations and a lack of clear mechanistic study. A plausible mechanism of this study will be later described in detail. Meanwhile, the DSC curves of electron-irradiated PAN fibers measured in an air atmosphere showed quite a different appearance compared with those obtained in the N_2_ atmosphere. Similar to measurements in N_2_, the DSC curve of non-irradiated PAN fiber showed a strong exothermic peak at 280.5 °C with a small tail below this temperature. As with the previous analysis, this result corresponds to the cyclization of the nitrile group induced by radical and ionic mechanisms. However, in contrast to the measurements in N_2_, a second peak at ~319 °C was apparently presented when measured in an air atmosphere. This exothermic reaction is well known as the oxidation reaction when heating of PAN fibers is conducted in air^49^, which is not seen when heated in N_2_. Similar to the N_2_ atmosphere, the T_onset_ shifted to lower temperature as the electron dose was increased. The detailed data are summarized in Table 1.

### Thermal stabilization and carbonization of electron-irradiated PAN fibers

The FT-IR spectra show the changes in chemical structure upon heating the electron-irradiated PAN fibers (Fig. 6). Upon heating non-irradiated PAN fibers, the vibration at 1593 cm^−1^ significantly increased, corresponding to the stretching of C=N. This result is attributed to the dehydrogenation and cyclization of PAN to form a polymer ladder structure^10^. To quantify the degree of stabilization, the extent of reaction (EOR) was calculated from the FT-IR absorption intensities of C=N and C≡N^29^. The EOR of the non-irradiated PAN fiber was calculated as 74.8%. As the electron dose increased to 200, 500, 1000 and 1500 kGy, the EOR gradually increased to 87.0, 88.8, 90.0, and 91.3%, respectively. These results verified that for a given heating condition, the electron irradiation prior to heat treatment can accelerate the cyclization reaction. This also indicates that electron irradiation on PAN fibers can effectively reduce the required heating time to achieve a certain degree of cyclization.

**Figure 7:**
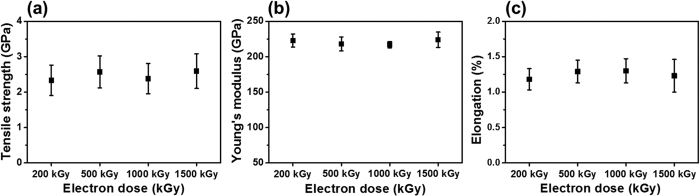
Mechanical properties of carbon fibers fabricated by electron irradiation of PAN fibers at various electron doses, followed by heat treatment at 230 °C for 30 min. (**a**) Tensile strength, (**b**) Young’s modulus, and (**c**) elongation.

After heat treatment of electron-irradiated PAN fibers, carbonization was conducted to convert the stabilized PAN fibers to carbon fibers. As expected, carbon fibers were not formed with non-irradiated PAN fibers by heat treatment at 230 °C for 30 min because of insufficient stabilization. However, carbon fibers were formed from electron-irradiated PAN fibers even though the fibers were thermally treated at 230 °C for only 30 min. The mechanical properties of carbon fibers fabricated from irradiated PAN fibers are shown in Fig. 7. Regardless of electron dose, the tensile strength, Young’s modulus, and elongation exceeded 2.3 GPa, 216 GPa, and 1.2%, respectively. These tensile properties are similar to those of carbon fibers fabricated from non-irradiated PAN fibers, which are heat treated at 230 °C for 120 min^51^. Therefore, electron beam irradiation on PAN fibers, even at a low electron dose of 200 kGy (irradiation time: 200 s), can effectively reduce (by 4-fold) the heat treatment time of the stabilization process.

### Mechanism study of electron-irradiated PAN fibers during stabilization process

**Figure 8:**
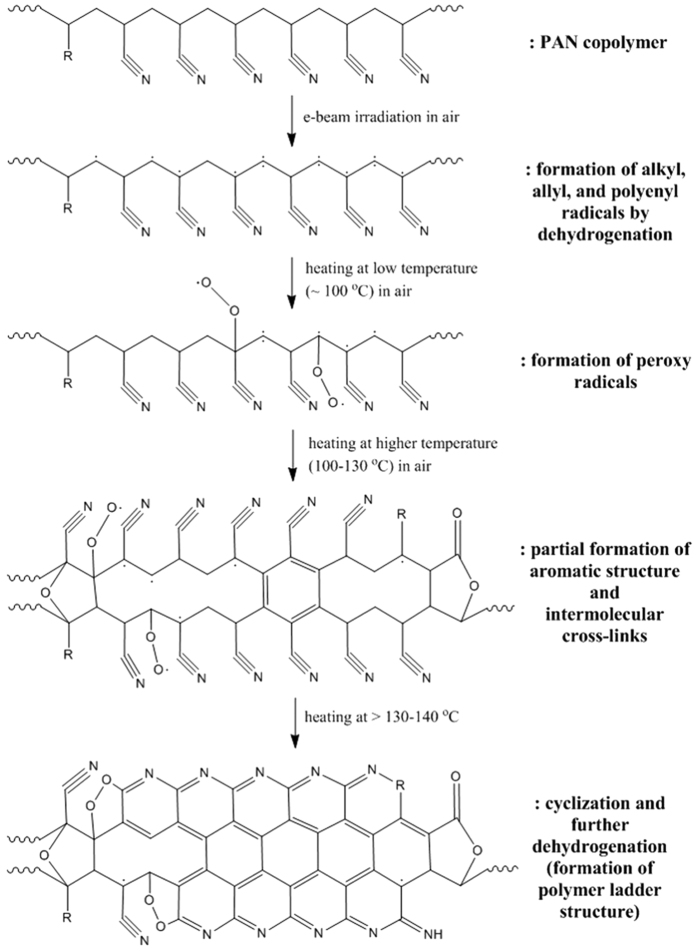
Suggested mechanism of thermal stabilization of electron-irradiated PAN fibers.

When energetic electrons are irradiated on PAN fibers, various free radicals can be formed by radiolysis of molecular bonds. In this study, alkyl, allyl and polyenyl radicals were readily formed on the PAN polymer chains by electron-beam irradiation. Because PAN is a semi-crystalline polymer with a T_g_ of ~100 °C, PAN is in a glassy state at room temperature, in which most of the polymer chains are fixed and immobilized within the crystalline lattice. When electron irradiation is conducted at low beam current density, the build-up of heat attributed to electron bombardment within the fiber is expected to be very low, which was verified in this study. Even at a relatively high dose (1500 kGy) of electron irradiation, no chemical reaction occurred in the fibers, although various radicals were formed. Therefore, we suppose that these radicals, which were formed on the polymer chains, were fixed and immobilized in the crystalline lattice and were inhibited to initiate any chemical reactions. In other words, the produced radicals were mostly preserved at the early stage of irradiation. These radicals further decayed or transformed to peroxy radicals under storage or heating in the presence of oxygen. After providing mobility and energy to these radicals, various reactions (cross-linking, dehydrogenation and cyclization) occurred as a result of radical recombination. Considering the stabilization process, the formation of peroxy radicals (CO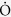) is supposed to be the key factor to shorten the thermal stabilization time of irradiated PAN fibers. In this study, electron-beam irradiation in air with low current density efficiently generated various radicals, and peroxy radicals (CO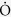) were gradually formed at low temperature (100 °C) during the stabilization process. These peroxy radicals are electrophiles, which can initiate the cyclization of nitrile groups by electrophilic attack of the carbon of the nitrile group. Therefore, the T_onset_ of cyclization shifted to lower temperature as the electron dose was increased because of the earlier initiation of cyclization by peroxy radicals prior to the conventional ionic and radical mechanism of PAN copolymer stabilization. As a result, the suggested stabilization mechanism, including various reactions of electron-beam irradiated PAN fibers, is shown in Fig. 8 through a comprehensive study based on our analysis.

## Conclusions

Electron-beam irradiation was performed on commercial PAN copolymer fibers prior to thermal stabilization. To solely investigate the irradiation effect on the thermal stabilization, irradiation was performed at low beam current density with sufficient cooling on the precursor fibers. As a result of electron irradiation, various radicals (alkyl, allyl, and polyenyl) were formed within the fibers. Typically, commercial PAN fibers are produced with high crystallinity and the polymer chains are oriented along the fiber axis. Therefore, the radicals produced on the polymer chains were trapped and immobilized in the crystalline lattice of the fibers with an oriented direction. Because of this trapping and immobilization, significant changes and chemical reactions were not observed within the fibers. Upon exposure to air or heating at low temperature (100 °C), the produced radicals intrinsically decayed but gradually transformed to peroxy radicals. These peroxy radicals seemed to play a significant role in shortening the thermal stabilization. The electrophilic peroxy radicals can initiate the cyclization of nitrile groups by electrophilic attack of the carbon of the nitrile group prior to the conventional ionic and radical mechanisms of the cyclization reaction. Therefore, during thermal stabilization of irradiated PAN fibers, the onset temperature of the exothermic cyclization reaction was shifted to lower values as the electron dose was increased. As a result, fast stabilization was achieved by heating the PAN fibers at 230 °C for 30 min, even for those irradiated at a low electron dose (200 kGy). Therefore, electron-beam irradiation (200 s) prior to thermal stabilization can effectively reduce the energy and time consumption of conventional thermal stabilization (120 min) by 4-fold.

## Materials and Methods

### Materials

Commercial PAN fibers were provided by Sinosteel Jilin Carbon Co., Ld (Jilin, China) as a precursor for carbon fibers. One tow of fibers is consisted to be 12k filaments. *N*, *N*-Dimethylformamide (DMF) was provided by Daejung Chemical & Metal Co., Ltd (≥99.5%, Republic of Korea) and was used as received.

### Electron-beam irradiation of PAN fibers

Electron-beam irradiation was conducted with 1 MeV beam energy and 1 mA beam current in air (EB Tech Co., Ltd., Republic of Korea). PAN fibers were placed and fixed on a water-cooled stage to prevent temperature rise during irradiation. No significant shrinkage of fibers was observed after irradiation. The irradiation area was 735 cm^2^, so the beam current density was calculated as 1.36 μA cm^−2^. The electron dose was controlled by changing the irradiation time to 200, 500, 1000 and 1500 s, which corresponds to 200, 500, 1000, and 1500 kGy, respectively. The uniformity of electron beam irradiation was guaranteed by the facility provided the irradiation service and reproducible results by our experiments.

### Thermal stabilization and carbonization of electron-irradiated PAN fibers

The electron-beam irradiated PAN fibers were stabilized by heat treatment at 230 °C for 30 min in air. Subsequently, carbonization was performed on the stabilized fibers at 1200 °C with a heating rate of 5 °C/min in an N_2_ atmosphere. Finally, the carbon fibers were fabricated.

### Characterization of electron-irradiated PAN, stabilized, and resultant carbon fibers

Fourier transform infrared (FT-IR) spectroscopy was performed on a Nicolet iS10 (Thermo scientific, USA) using the attenuated total reflectance (ATR) mode in the range of 4000–750 cm^−1^. The FT-IR spectra were obtained over 32 scans at a resolution of 3.857 cm^−1^. All of the fibers were dried at 60 °C in air for at least 24 h prior to measurement to minimize the adsorbed water on the sample surface. X-ray diffraction (XRD) measurements were conducted on a SmartLab (Rigaku, Japan) with Cu Kα radiation (λ = 1.54 Å). The 2θ value was scanned from 10 to 60° at a scan speed of 4°/min. Electron spin resonance (ESR) spectroscopy was performed on a JES-FA100 (JEOL, Japan). The microwave frequency was 9.11 GHz with modulation of 100 kHz and the power was 0.998 mW. The spectra were recorded by changing the external magnetic field with a sweep time of 20 s, sweep width of 250 mT, and modulation width of 0.01 mT. The gel contents of the irradiated fibers were evaluated by dissolving in DMF for 24 h at 60 °C, followed by drying for 12 h at 60 °C in a vacuum. The gel contents were calculated by dividing the mass of residual gel after dissolving to the initial mass of the fiber before dissolving in DMF^24^. Differential scanning calorimetry (DSC) was performed on an Auto Q20 (TA Instruments, USA) in N_2_ and air atmospheres at a heating rate of 5 °C/min. The mechanical properties of carbon fibers were measured on an automatic individual fiber test system (FAVIMAT+, Textechno, Germany). The gauge length and test speed were 25 mm and 2 mm/min, respectively. Fifteen specimens were measured for each sample.

## Additional Information

**How to cite this article**: Park, S. *et al*. Comprehensive stabilization mechanism of electron-beam irradiated polyacrylonitrile fibers to shorten the conventional thermal treatment. *Sci. Rep.* **6**, 27330; doi: 10.1038/srep27330 (2016).

## Supplementary Material

Supplementary Information

## Figures and Tables

**Table 1:** Characterization of DSC curves of PAN fibers irradiated at various electron doses.

**Dose (kGy)**	**Atmosphere**	**T**_**onset**_^a^ (^**o**^**C)**	**T**_**p,1**_^b^ (^**o**^**C)**	**T**_**p,2**_^c^ (^**o**^**C)**	**Atmosphere**	**T**_**onset**_ (^**o**^**C)**	**T**_**p,1**_ (^**o**^**C)**	**T**_**p,2**_ (^**o**^**C)**
0	N_2_	174.2	–^d^	277.2	air	173.2	280.5	318.8
200		137.1	–^d^	276.4		146.4	266.6	319.6
500		134.4	–^d^	279.1		144.4	255.8	318.8
1000		133.4	250.9	281.0		135.6	245.9	319.1
1500		132.2	242.9	283.0		129.7	240.4	318.4

^a^T_onset_ : onset temperature of exothermic reaction.

^b^T_p,1_ : first exothermic peak temperature.

^c^T_p,2_ : second exothermic peak temperature.

^d^First peak temperature not distinguishable from second peak temperature.
