# Supplementary material for: Comprehensive stabilization mechanism of electron-beam irradiated polyacrylonitrile fibers to shorten the conventional thermal treatment
Source: Sci Rep. 2016 Jun 28;6:27330. doi: 10.1038/srep27330 (PMC4923868; doi:10.1038/srep27330)
Supplement: Supplementary Information [file srep27330-s1.pdf]

Supporting Information

Comprehensive stabilization mechanism of electron-beam irradiated polyacrylonitrile fibers to shorten the conventional thermal treatment

Sejoon Park¶, Seung Hwa Yoo¶, Ha Ri Kang, Seong Mu Jo, Han-Ik Joh, Sungho Lee*

Carbon Convergence Materials Research Center, Institute of Advanced Composite Materials, Korea Institute of Science and Technology, 92, Chudong-ro, Bongdong-eup, Wanju-gun, Jeollabuk-do 565-905, Republic of Korea

*e-mail: sunghol@kist.re.kr

¶ Authors contributed equally to this work

Analyses of electron-beam irradiated PAN fibers

When an electron-beam is irradiated on PAN fibers, various radicals are formed. These radicals show specific hyperfine structures with a splitting constant and line-width when observed by ESR. Therefore by ESR spectra analysis, one can identify the radical species which is presented in the irradiated material. Usually, the spectra of these radicals are superimposed which results as a spectrum showed in Figure S1a. By analyzing the spectrum in Figure S1a, the hyperfine structure is estimated to be as Figure S1b. In detail, the hyperfine splitting of 1.4 mT (a_1), 2.8-3.6 mT (a_2) and 1.9-2.2 mT (a_3) with line-widths of 0.7-1.5 mT were observed. Based on the hyperfine splitting constants of previous reports (Figure S2), it is estimated that a_1 is originated from the broad singlet structure of polyenyl radical. Furthermore, a_2 is estimated to be originated from the five-line hyperfine structure of alkyl radical. Finally, the six- and ten-line hyperfine structures with splitting constant a_3 are estimated that it comes from the alkyl and allyl radicals.

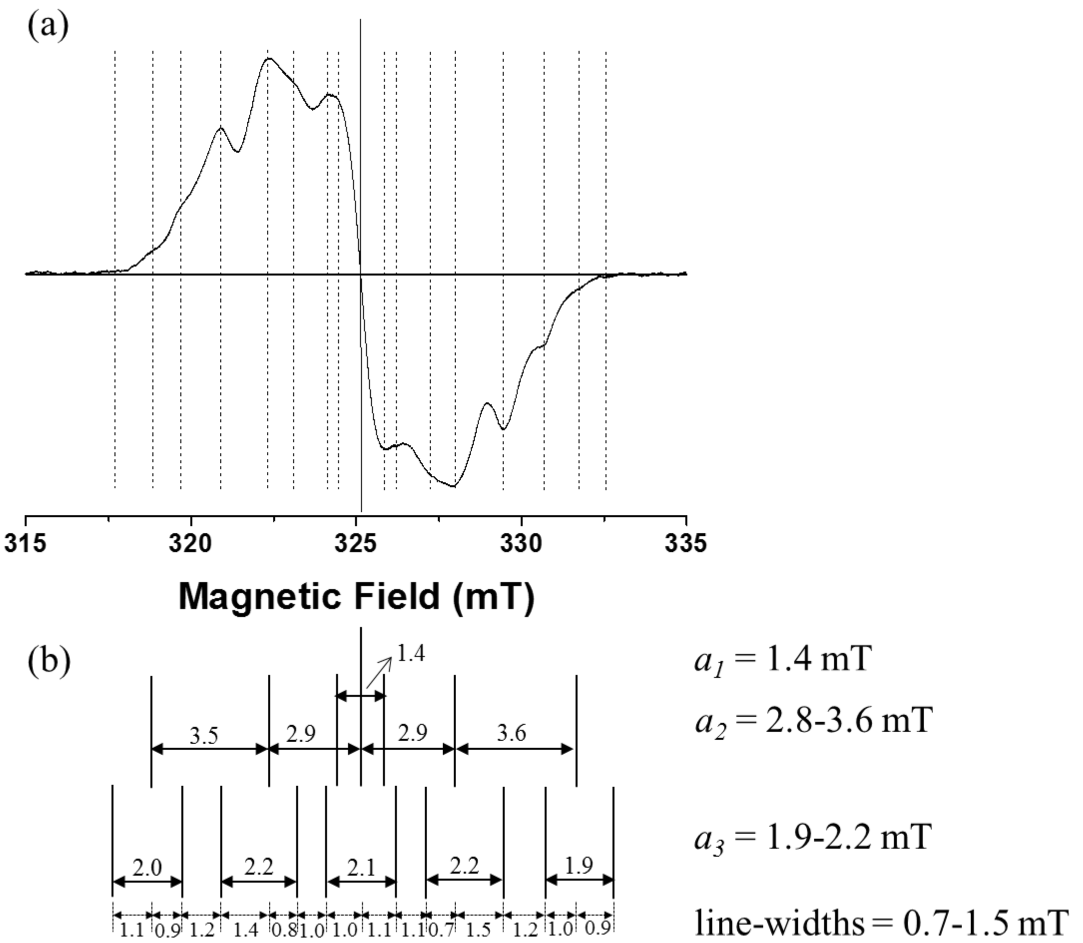

Figure S1 (a) ESR spectrum of PAN fibers irradiated at 200 kGy electron dose. (b) Estimated hyperfine structure of (a).

Type	Structure	ESR hyperfine structure & splitting constant (*a*)
Alkyl	(1) 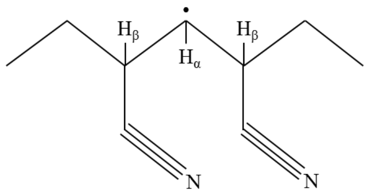	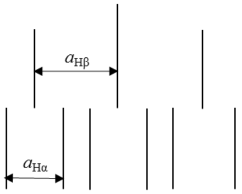 $a_{H\beta}$ = 2.8- 3.3 mT<br>$a_{H\alpha}$ =1.3-2.2 mT<br>six-line hyperfine spectrum
	(2) 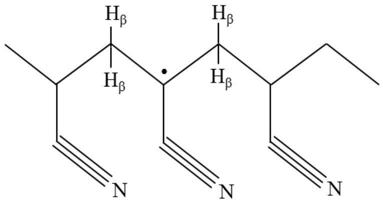	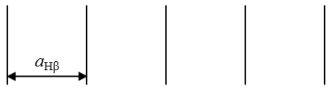 $a_{H\beta}$ = 2.8- 3.3 mT<br>five-line hyperfine spectrum
Allyl	(3) 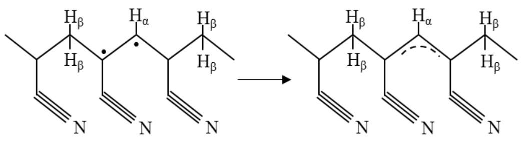	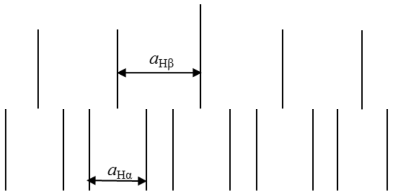 $a_{H\alpha}$, $a_{H\beta}$ similar with alkyl<br>ten-line hyperfine spectrum
Polyenyl	(4) 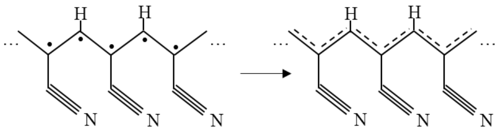	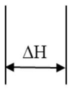 ΔH = 0.89 - 2.7 mT<br>broad singlet spectrum

Figure S2. The types of radicals in electron-irradiated PAN fibers with its corresponding molecular structures and ESR hyperfine structures.

The change of relative radical concentration for irradiated-PAN fibers at 200 kGy was observed during storage in air (Figure S3). The radical concentration seemed to exponentially decrease as the storage time in air increased. This phenomenon is known as the intrinsic decay of radicals which are commonly observed for gamma ray irradiation on polymers [1-3]. In this study, the radical concentration decreased to 65%, 58%, 17%, and 3% to its initial value (3 h after irradiation) at 20, 30, 143, 193 h storage in air after irradiation.

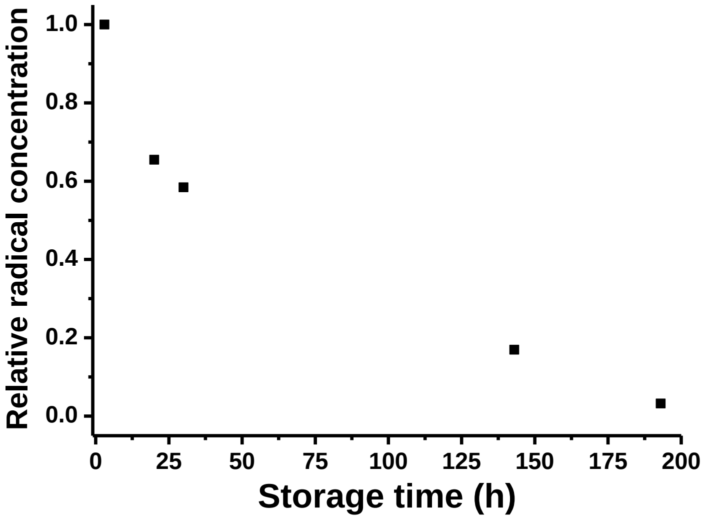

Figure S3. Relative radical concentration of irradiated-PAN fibers at 200 kGy during storage in air.

Along with the decrease in radical concentration, the g-value of the presented radicals gradually shifted to higher values. It has been reported that oxidized radicals have higher g-value than the non-oxidized ones, implying that gradual increase in g-values can be caused by gradual change of initially formed radicals to peroxy radicals. According to the observation, the g-value

gradually increased from its initial value as the storage time increased (Figure S4). Furthermore, the g- value also increased when the irradiated-PAN fiber was heated at 100 ^{o}C. By increasing the heating time as 10, 20, 30 min, the g-value increased as 2.00286 ± 0.00014, 2.00306 ± 0.00009, 2.00299 ± 0.00020, respectively, compared to its initial value 2.00268 ± 0.00011. This indicates that the radicals produced by irradiation are gradually changing to peroxy radicals as they are exposed in air. This is also verified by the ESR spectra that the hyperfine structure is transformed to a broad singlet structure.

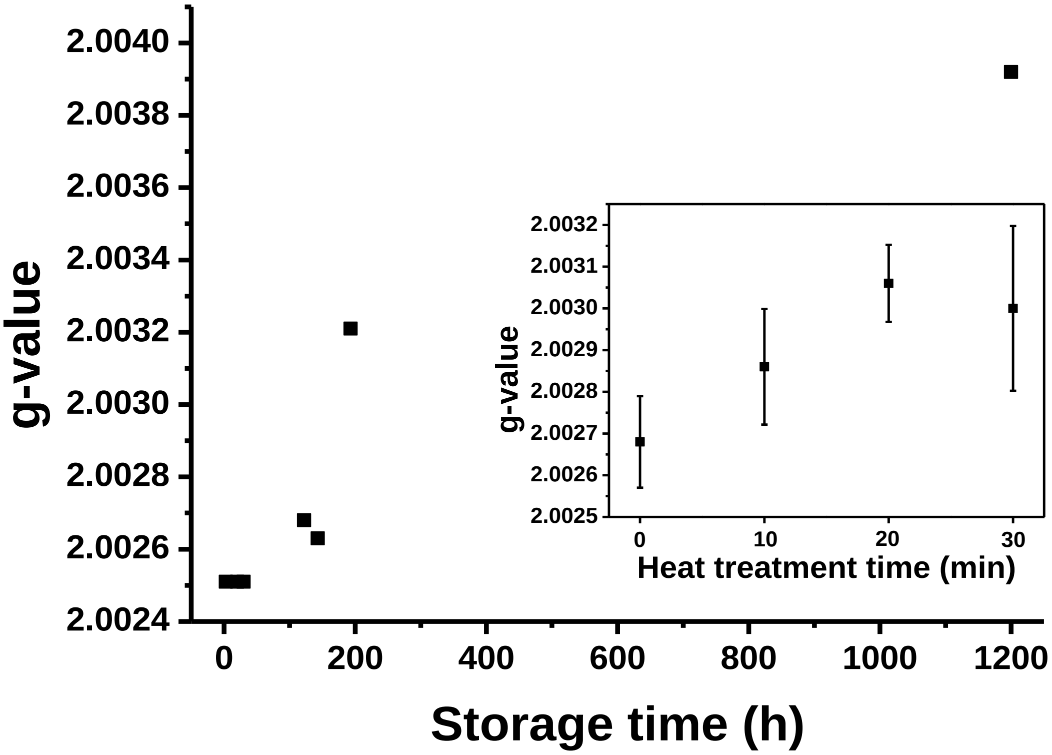

Figure S4. Variation of g-value of irraditated-PAN fibers at 200 kGy during storage, and (inset) heat treatment at 100 ^{o}C for different time in air.

For complementary analysis, the oxygen content of irradiated-PAN fibers were checked by elemental analysis (Figure S5). The weight percent of oxygen increased from 2.110 ± 0.184, 2.355 ± 0.028, 2.543 ± 0.011 for 0, 20, 1500 kGy electron dose, respectively, which was measured after 6 days from irradiation. In case of measurement after 53 days from irradiation, the non-irradiated (0 kGy) PAN fiber contained 1.993 ± 0.086 % oxygen, which is within the error of measurement after 6 days from irradiation. As the electron dose increased from 200 to 1500 kGy, the oxygen content increased as 2.352 ± 0.017 to 2.680 ± 0.026, respectively. These results indicate that oxygen content increased in the fiber as the storage time or electron dose was increased. These facts well match with our analysis that oxygen is attached to the radicals to form peroxy radicals in irradiated-PAN fibers.

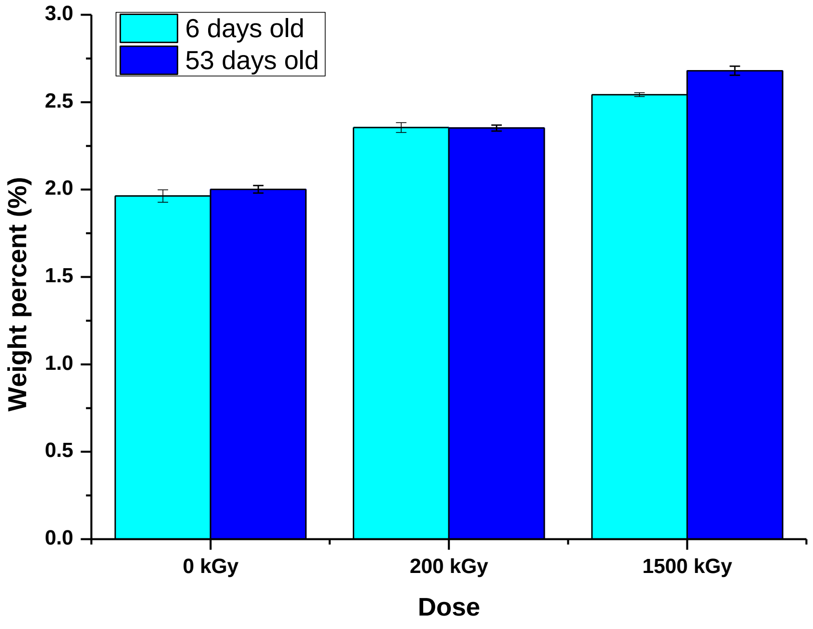

Figure S5. Oxygen contents of irraditated-PAN fibers after storage of 6 days and 53 days in air.

The FT-IR spectra of irradiated-PAN fibers at 200 kGy followed by heat treatment at 110, 120, 130 °C for 30 min are shown in Figure S6. As the heating temperature gradually increased from 110 to 130 °C, the vibrations of conjugated C-C bonds (1614 cm^{-1}) originated from electron-beam irradiated PAN fiber gradually shifted to lower wavenumber of 1590 cm^{-1}. This indicates that the conjugated C-C bonds on polymer chains are gradually changed to a ring structure by merging with neighbored chains. Along with the ring formation, acid C=O bonds emerged, which is expected as a result of ring structure formation.

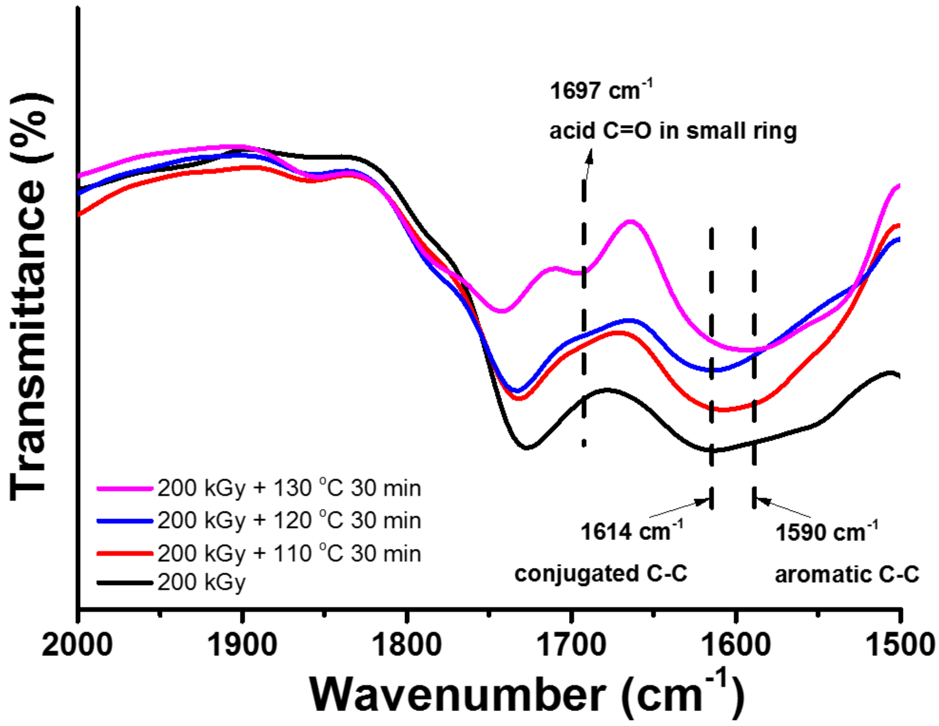

Figure S6. FT-IR spectra of irradiated-PAN fiber at 200 kGy, followed by heating at 110, 120, 130 °C for 30 min in air.

The scanning electron microscope images of non-irradiated and irradiated-PAN fibers are shown in Figure S7. Electron irradiation induced no significant changes in the morphology of PAN fibers.

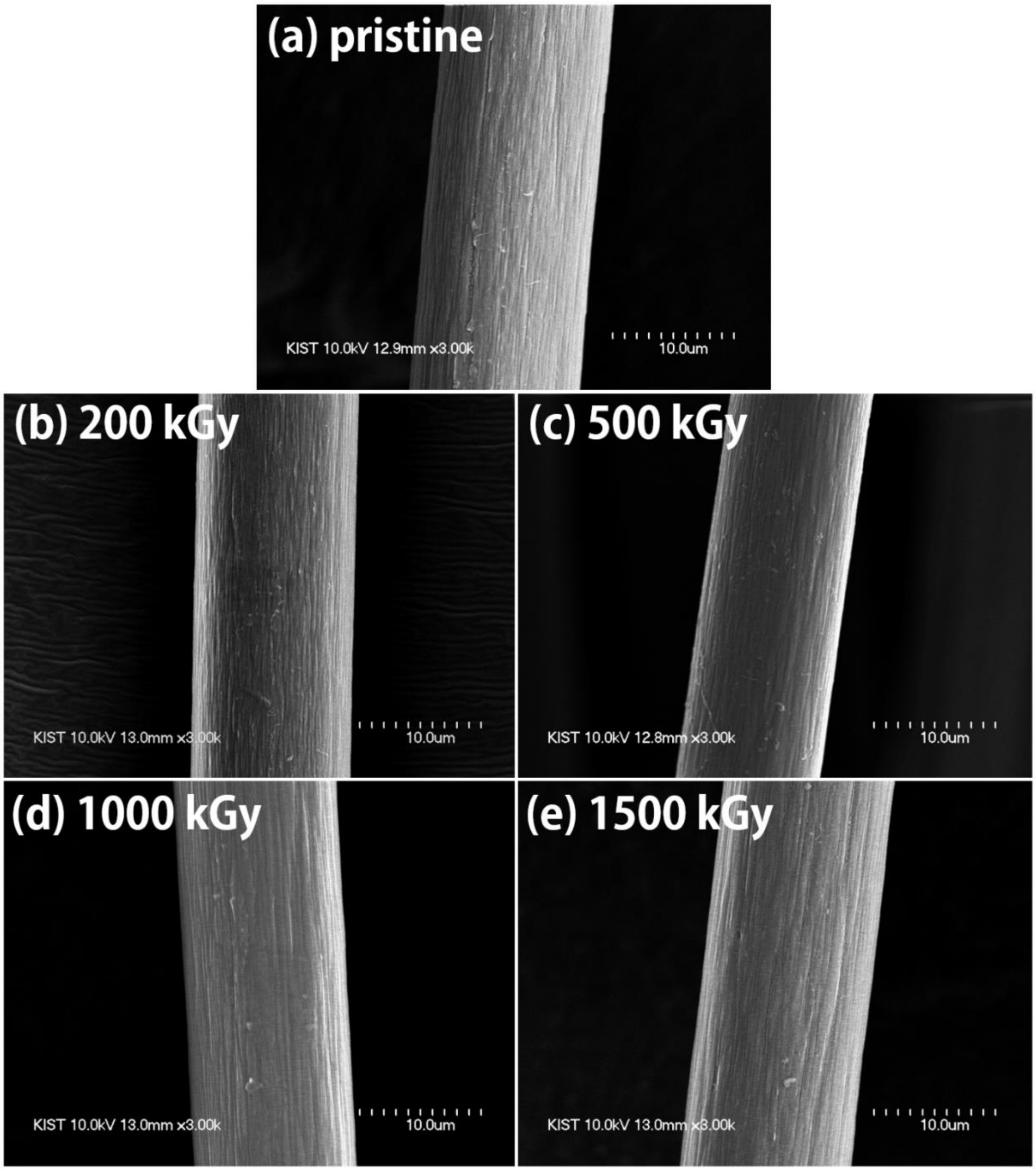

Figure S7. Scanning electron microscope images of PAN fibers. (a) pristine, irradiated at (b) 200 kGy, (c) 500 kGy, (d) 1000 kGy, and (e) 1500 kGy.

The energy consumption was calculated based on the power and duration of each electron beam irradiation and conventional heating process. In case of electron beam irradiation and thermal stabilization, the energy consumption was calculated as 477.2 kJ (1. power of electron beam: 1 MeV × 1mA = 1 kW, 2. energy consumption: 1 kW × 200 s = 200 kJ, 3. power of conventional heating: 220 V × 0.7 A = 154 W, 4. energy consumption: 154 W × 1800 s = 277.2 kJ, 5. total energy consumption: 200 kJ + 277.2 kJ = 477.2 kJ). On the other hand, the energy consumption of conventional thermal stabilization was calculated as 1108.8 kJ (154 W × 7200 s). From this fact, the energy could be saved as 57% by electron beam irradiation prior to thermal stabilization. Therefore, it is conclusive that electron beam irradiation is effective to lower the production cost of carbon fibers by saving energy and time consumption.

References:

[1] Silva P, Albano C, Perera R, Gonzalez J, Ichazo M. An electron paramagnetic resonance study of PP and PP/SBS blends irradiated with gamma rays. Nucl. Instr. Meth. Phys. Res. B 2004;226:320-326.

[2] Loy BR. Electron Spin Resonance Studies of Free Radical Decay in Gamma-Irradiated Polyethylene. J. Polym. Sci. 1960;44:341-347.

[3] Zhao Y, Wang M, Tang Z, Wu G. ESR study of free radicals in UHMW-PE fiber irradiated by gamma rays. Radiat. Phys. Chem. 2010;79:429-433.
